# Association between Angiotensinogen M235T Polymorphism and Preeclampsia in Iranian Pregnant Women

**Published:** 2014-12

**Authors:** Raha Afshariani, Jamshid Roozbeh, Maryam Sharifian, Mahbobeh Ghaedi, Alamtaj Samsami Dehaghani, Abbas Ghaderi

**Affiliations:** 1 Department of Public Health, School of Health and Nutrition, Shiraz University of Medical Sciences, Shiraz, Iran; 2 Urology Nephrology Research Centre, Shiraz University of Medical Sciences, Shiraz, Iran; 3 Department of Neurology, Student Research Committee, Shiraz University of Medical Sciences, Shiraz, Iran; 4 Department of Obstetrics and Gynecology, Shiraz University of Medical Sciences, Shiraz, Iran; 5 Shiraz Institute of Cancer Research, Shiraz University of Medical Sciences, Shiraz, Iran

**Keywords:** Angiotensinogen M235T Polymorphism, Preeclampsia

## Abstract

**Objective:** To determine the possible association between the M235T variant of angiotensinogen gene and preeclampsia in Iranian preeclamtic women with hypertension during pregnancy.

**Materials and methods:** During a case control study, we used polymerase chain reaction-based restriction fragment length polymorphism (PCR-RFLP) analysis to investigate the association between M235T polymorphism in preeclamtic women compared to normotensive controls.

**Results:** The M235T polymorphism was significantly associated with increased preeclampsia risk in the studied population as supported by a p value of 0.017 and chi-square value of 8.12. The frequency of mutated allele and genotype distribution showed a significant difference between preeclamtic women and control groups.

**Conclusion:** The result indicates that the AGT M235T polymorphism plays a significant role in preeclampsia observed in selected Iranian preeclamtic women, and it can be considered as a major risk factor for preeclampsia.

## Introduction

Preeclampsia is a serious disease associated with human pregnancy and occurs in about 5–7% of all pregnancies which remains a of maternal and fetal morbidity and mortality (1,2). It is characterized by hypertension (systolic blood pressure higher than 140 mm Hg and/or diastolic blood pressure higher than 90 mm Hg), proteinuria and edema after the 20th week of gestation. In addition, other modifications such as platelet count < 100,000/mm3 and elevated serum aminotransferase concentration might be observed (3). Epidemiological and family based studies in several geographically and ethnically distinct populations indicate that preeclampsia is a multifactorial disorder with a familial tendency, while it is influenced by race, parity, health status of placenta, diet and body size (4-6). Recently, there are several studies exploring the mechanisms of preeclampsia. As there is a complex interaction between a variety of genetic and environmental factors in preeclampsia, the precise cause of preeclampsia has not been determined (7).

The renin–angiotensin system (RAS) has an important role in blood pressure regulation during pregnancy. It is suggested that alterations in the RAS play a significant role in the pathophysiology of preeclampsia (4, 8).

Therefore, the products of genes involving the components of this system can be a potential candidate for preeclampsia and hypertension during pregnancy. It has been suggested that some genetic factors such as gene polymorphisms of the RA system may has an important role in the regulation of blood pressure (BP) especially in preeclampsia and may contribute to the heterogeneity of preeclampsia in different ethnic groups (9).

Angiotensinogen (AGT) is an important component of the RA system, which is a powerful regulatory system with an effective influence on salt and water metabolism and blood pressure. AGT is the precursor protein to angiotensin II, which plays a primary role in the regulation of blood pressure by the renin–angiotensin system (9). The M235T angiotensinogen gene mutation is a single base pair substitution of thymine (T) with cytosine (C) at nucleotide 704 (T704C) in exon 2 of the angiotensinogen gene (chromosome 1q42- 43), leading to the substitution of methionine with threonine at amino acid position 235 in the pre proangiotensinogen molecule (M235T). In addition, T235 allele represents the mutant allele, while M235 allele represents the wild type (10, 11).

Different studies have showed that a variant of AGTT235 is associated with preeclampsia in the Caucasian (12) and in the Japanese women (13). Other studies have also reported familial tendency of hypertension in Korean women; furthermore, association between AA genotype of angiotensinogen gene and high blood pressure (BP) leads to hypertensive disorders in pregnancy (7). On the contrary, Guo et al. (14) showed that there was no significant association between M235T allele of angiotensinogen and preeclampsia/eclampsia in a population case-control study among the Australians and also Chinese women (14, 15) 

The aim of this study was to analyze the occurrence of M235T gene polymorphism in Iranian pregnant women with preeclampsia as compared to normotensive pregnant woman to investigate the association between M235Tpolymorphism and preeclampsia.

## Materials and methods

***Participants***

During a case control study in Hafez Hospital, a university hospital of Shiraz University of Medical Sciences, We studied 69 pregnant women with preeclampsia. The mean age of patients was 27.7 years. The definition used for preeclampsia is based on hypertension (systolic BP is greater than 140mmHg, or diastolic BP is greater than 90 mmHg) with proteinuria>300 mg/l in a random specimen or an excretion of>300 mg per 24 hours after 20 weeks of gestation in a woman who was normotensive before pregnancy (16). Our findings confirmed the absence of proteinuria for more than four weeks after delivery which was applied as a main factor to roll out underlying renal disease. Other exclusion criteria in this study were overt cardiac disease, serum creatinine >150 mol/L, and the presence of other systemic disease.

The control group, recruited from the same center, consisted of 103 normotensive volunteer pregnant women with at least two pregnancies and no history of preeclampsia. The mean age of control group was 25.8 years. The Ethics Committee of Motahari Hospital approved the protocol and informed consent was obtained from all participants

***DNA extraction***

DNA was extracted from the peripheral lymphocytes by salting out method as described by Miller et al. (1998) (16).

***Mutation detection***

The presence of M235T polymorphism within angiotensinogen gene was analyzed using polymerase chain reaction–based restriction fragment length polymorphism (PCR-RFLP). The M235T is a single base pair substitution of thymine (T) with cytosine (C) at nucleotide 704 of the angiotensinogen gene, which leads to the substitution of methionine with threonine at amino acid position 235 in the pre–proangiotensinogen molecule (M235T), which creates a restriction site for *PSYI*. To detect the M235T transition in angiotensinogen gene, ~100 ng of DNA was used for PCR amplification. The PCR was carried out in a total volume of 50μ1, (containing 

50 ng of the forward primer

     5’CTTGGGGAGCTGAAGGACTACTAC3’ and 

50 ng of the reverse primer

     5’CACTTTGTGACCATTCCGGTTTG3’ 200 μM 

each dNTP), 10mM Tris-Hcl

     (PH = 8.3), 50mM KCL, 3mM Mgcl2 and 1 unit 

Taq polymerase. 

     5’CTTGGGGAGCTGAAGGACTACTAC3’, 50 ng reverse primer 5’CACTTTGTGACCATTCCG GTTTG3’, 200 μM each dNTP, 10mM Tris-Hcl (PH = 8.3), 50mM KCL, 3mM Mgcl2 and 1 unit Taq polymerase.

PCR parameters for detection of the M235T transition were as follows: an initial denaturation step of 5 minutes at 95°C followed by 32 cycle of 95°C / 60 seconds (denaturation), 61°C/60 seconds (annealing) and 72°C/60 seconds (extension), as well as final extension for 7 minutes at 72°C to ensure a complete extension of all PCR products. The amplified PCR fragment of 165 bp was digested overnight at 37^º^ C with restriction enzyme PSYI followed by electrophoresis on 3 % agarose gel. The mutated homozygous variant (235TT) produced 2 fragments of 141bp and 24bp. While heterozygote (M235T) produced 3 fragments of141bp, 24bp and 165bp, while wild-type (235MM) produced 1 fragment of 165bp.

***Statistical analysis***

Descriptive values were expressed as the mean ± SD. Allele frequencies were calculated for each genotype by allele counting. Comparison of allele frequencies between case and control groups were determined using chi-square test using SPSS for windows version 10 (SPSS Inc., Chicago. Illinois, USA). Fisher’s exact test was used when the number of observations in any group was ≥5. A value of p < 0.05 was considered significant.

## Results

In this study, 69 women with singleton pregnancy complicated by preeclampsia and 103 control subjects were genotyped for common mutation M235T. The mean age values of the cases and control were 27.7 and 25.8 year, respectively. The frequencies of MM, MT and TT genotypes were 44.9%, 36.2 % and 18.8% in preeclampsia cases, and 66%, 19.4 % and 14.6% in the controls. The frequency of mutated allele (T) and normal allele (M) in patient and control groups were 36%, 24%, 64 % and 76%, respectively (Table 1). The genotype distribution of the AGT M235T polymorphism differed significantly among preeclamptic women. As compared to the control individuals, the frequency of homozygotes for the T allele was significantly higher in preeclamptic women. The difference between the two groups was not significant (χ 2 = 8.12; p = 0.017). Table 1 shows the comparison of the prevalence of the M235T polymorphism between women with preeclampsia and normal controls.

## Discussion

Preeclampsia is characterized by hypertension (blood pressure R140 mm Hg systolic and/or R90 mm Hg diastolic), new-onset proteinuria, or edema after the 20th week of gestation. This heterogeneous disorder occurs in 5-7% of all pregnancies, remains a cause of maternal and fetal mortality, and is considered as an important economic healthcare burden worldwide (1,9).

It may be caused by poor placental perfusion, may be due to incomplete invasion of fetal trophoblast cells into the uterus of the mother and maternal resistance against this invasion. So, both mother and fetus contribute to the risk (17-20). Many risk factors (genetic, nutrition, immunologic, and infectious origins) and their pathologic mechanisms (abnormal placentation, oxidative stress, and endothelial dysfunction) have been proposed, yet this disorder has remained as a ‘‘disease of theories’ (21). The absence of stimulation of the RAS in women developing hypertension during pregnancy, or in women with preeclampsia has been confirmed by many studies. There are several evidences showing that the plasma renin activity, plasma rennin concentration, angiotensin II, angiotensinogen and plasma urinary aldosterone levels are all lower in preeclampsia as compared with normotensive pregnant women because the RAS plays an integral role in the pathophysiology of preeclampsia. Also, it is shown that the genetic variants may play a role in the development of preeclampsia (22, 23).

**Table 1:** Comparison of distribution of M235T genotype and allele's frequencies between preeclamtic women and normal control groups

	MM	MT	TT	M[%]‍	T[%]
**Preeclamtic women**	**44.9**	**36.2**	**18.8**	**36**	**64**
**Normal control**	**66**	**19.4**	**14.6**	**24**	**76**

Heterozygous MT, Homozygous mutant T/T, Normal MM

Human linkage and several genetic studies have showed the presence of polymorphisms in genes encoding key components of the RAS which account for the proportion of the variance inactivity of the system. Studies of these polymorphisms have reported both positive and negative association with preeclampsia (24- 27). Jeunemaitre et al. showed that increased concentration of plasma or tissue angiotensinogen in women with variants of angiotensinogen, (for example M235T), can lead to increased baseline or reactive production of angiotensin II, which is final effector hormone of the RA system. 

This continues over-stimulation can activate auto regulatory mechanisms, resulting in increased vascular tone and also hypertrophy. This condition can aggravate an imbalance mechanism between vasodilatory and vasoconstrictory factors which increases sensitivity to angiotensin II and reduces the plasma levels of most components of this system (23, 24,27, 28). Studies by Ward et al. (12) and Arngrimsson et al. (29) showed that a molecular variant of angiotensinogen (M235T) in the Caucasians and Japanese women is significantly associated with preeclampsia, so influences the development of this disorder. 

In Korean females, familial tendency of hypertension and angiotensinogen AA genotype had an association with high BP in hypertensive disorder during pregnancy (14). 

On the other hand, Guo et al. (15) found that there was no significant correlation between the M235T allele of angiotensinogen and preeclampsia/eclampsia in a population case-control study among the Australians and Chinese, but this may be attributable to the criteria used for diagnoses. The association between M235T polymorphisms and preeclampsia was studied in pregnant women with preeclampsia and normotensive volunteer pregnant women. 

It was speculated that a possible link exists between this polymorphism and the occurrence of preeclampsia in the studied population. A significant association was observed between M235T polymorphism and preeclampsia. The genotype distribution and frequency of mutated allele showed significant differences between preeclamptic woman and control groups. The frequency of mutated T allele was higher in preeclamtic women than normal control group (χ 2 = 8.12; p = 0.017)

However, this is a preliminary study and requires more hypertensive and normotensive pregnant women to be comparatively studied in order to improve the statistical significance. In conclusion, our findings is in line with several studies in which they have suggested that the presence of M235T angiotensinogen gene polymorphism should be considered and investigated as a risk factor in women with preeclampsia. In these cases, it is recommended that the arterial blood pressure should be monitored, and all the possible causes of hypertension during pregnancy should be tested, including genetic analysis of M235T variant of the angiotensinogen gene.

## References

[1] Cooper DW, Brennecke SP, Wilton AN (1993). Genetics of preeclampsia. Hypertension Pregnancy.

[2] Roberts JM, Pearson G, Cutler J, Lindheimer M, NHLBI Working Group on Research on Hypertension During Pregnancy (2003). Summary of the NHLBI Working Group on Research on Hypertension During Pregnancy. Hypertension.

[3] Qureshi AI, Frankel MR, Ottenlips JR, Stern BJ (1996). Cerebral hemodynamics in preeclampsia and eclampsia. Arch Neurol..

[4] Bouba I, Makrydimas G, Kalaitzidis R, Lolis DE, Siamopoulos KC, Georgiou I (2003). Interaction between the polymorphisms of the renin-angiotensin system in preeclampsia. Eur J Obstet Gynecol Reprod Biol.

[5] Sutherland A, Cooper DW, Howie PW, Liston WA, MacGillivray I (1981). The incidence of severe preeclampsia amongst mothers and mothersin-law of preeclamptic and controls. Br J Obstet Gynaecol.

[6] Gant N, Gilstrap (1996). Hypertension in pregnancy. ACOG. January Technical Bulletin 219.

[7] Kim YJ, Park MH, Park HS, Lee KS, Ha EH, Pang MG (2004). Associations of polymorphisms of the angiotensinogen M235 polymorphism and angiotensin-converting-enzyme intron 16 insertion/deletion polymorphism with preeclampsia in Korean women. Eur J Obstet Gynecol Reprod Biol.

[8] Broughton PF, Rubin PC (1988). The renin angiotensin system in normal and hypertensive pregnancies. Handbook of hypertension.

[9] Choi H, Kang JY, Yoon HS, Han SS, Whang CS, Moon IG, Et al (2004). Association of Angiotensin-converting enzyme and angiotensinogen gene polymorphisms with preeclampsia. J Korean Med Sci.

[10] Russ AP, Maerz W, Ruzicka V, Stein U, Gross W (1993). Rapid detection of the hypertension-associated Met235 Thr allele of the human angiotensinogen gene. Hum Mol Genet.

[11] Jeunemaitre X, Charru A, Chatellier G, Dumont C, Sassano P, Soubrier F (1993). M235T variant of the human angiotensinogen gene in unselected hypertensive patients. J Hypertens Suppl.

[12] Ward K, Hata A, Jeunemaitre X, Helin C, Nelson L, Namikawa C (1993). A molecular variant of angiotensinogen associated with preeclampsia. Nat Genet.

[13] Arngrímsson R, Sigurõardóttir S, Frigge ML, Bjarnadóttir RI, Jónsson T, Stefánsson H (1999). A genome-wide scan reveals a maternal susceptibility locus for pre-eclampsia on chromosome 2p13. Hum Mol Genet.

[14] Guo G, Wilton AN, Fu Y, Qiu H, Brennecke SP, Cooper DW (1997). Angiotensinogen gene variation in a population case-control study of preeclampsia/ eclampsia in Australians and Chinese. Electrophoresis.

[15] Shim SS, Shim JY, Lim JH, Park JS, Jun JK, Bai KB (2003). Molecular biological approach to find out the etiology of hypertensive disorder in pregnancy: analysis of the polymorphism in the promoter of human angiotensinogen gene in Korean population. Korean J Obstet Gynecol.

[16] (1990). National High Blood Pressure Education Program Working Group Report on High Blood Pressure in Pregnancy. Am J Obstet Gynecol.

[17] Miller SA, Dykes DD, Polesky HF (1988). A simple salting out procedure for extracting DNA from human nucleated cells. Nucleic Acids Res.

[18] Roberts JM, Taylor RN, Musci TJ, Rodgers GM, Hubel CA, McLaughlin MK (1989). Preeclampsia: an endothelial cell disorder. Am J Obstet Gynecol.

[19] Shanklin DR, Sibai BM (1989). Ultrastructural aspects of preeclampsia. I. Placental bed and uterine boundary vessels. Am J Obstet Gynecol.

[20] Begum MR, Akhter S, Begum A, Khatun M, Quadir E, Choudhury SB (2002). Conservative management of eclampsia and severe pre-eclampsia--A Bangladesh experience. Medscape Womens Health.

[21] Kupferminc MJ, Peri H, Zwang E, Yaron Y, Wolman I, Eldor A (2000). High prevalence of the prothrombin gene mutation in women with intrauterine growth retardation, abruptio placentae and second trimester loss. Acta Obstet Gynecol Scand.

[22] Mignini LE, Villar J, Khan KS (2006). Mapping the theories of preeclampsia: the need for systematic reviews of mechanisms of the disease. Am J Obstet Gynecol.

[23] August P, Lenz T, Ales KL, Druzin ML, Edersheim TG, Hutson JM (1990). Longitudinal study of the renin-angiotensin-aldosterone system in hypertensive pregnant women: deviations related to the development of superimposed preeclampsia. Am J Obstet Gynecol.

[24] Mutter WP, Karumanchi SA (2008). Molecular mechanisms of preeclampsia. Microvascular Research.

[25] Jeunemaitre X, Soubrier F, Kotelevtsev YV, Lifton RP, Williams CS, Charru A (1992). Molecular basis of human hypertension: role of angiotensinogen. Cell.

[26] Rigat B, Hubert C, Alhenc-Gelas F, Cambien F, Corvol P, Soubrier F (1990). An insertion/deletion polymorphism in the angiotensin I-converting enzyme gene accounting for half the variance of serum enzyme levels. J Clin Invest.

[27] Morgan T, Craven C, Lalouel JM, Ward K (1999). Angiotensinogen Thr235 variant is associated with abnormal physiologic change of the uterine spiral arteries in first-trimester decidua. Am J Obstet Gynecol.

[28] Tamura T, Johanning GL, Goldenberg RL, Johnston KE, DuBard MB (1996). Effect of angiotensin-converting enzyme gene polymorphism on pregnancy outcome, enzyme activity, and zinc concentration. Obstet Gynecol.

[29] Arngrímsson R, Purandare S, Connor M, Walker JJ, Björnsson S, Soubrier F (1993). Angiotensinogen: a candidate gene involved in preeclampsia?. Nat Genet.

